# Risk Factors and Outcomes of Pulmonary Hypertension in Infants With Bronchopulmonary Dysplasia: A Meta-Analysis

**DOI:** 10.3389/fped.2021.695610

**Published:** 2021-06-25

**Authors:** Ying Chen, Di Zhang, Ying Li, Aixia Yan, Xiaoying Wang, Xiaoming Hu, Hangting Shi, Yue Du, Wenhui Zhang

**Affiliations:** ^1^Department of Neonatology, Children's Hospital Affiliated to Capital Institute of Pediatrics, Beijing, China; ^2^Department of Pediatrics, Aerspace Central Hospital, Beijing, China

**Keywords:** bronchopulmonary dysplasia, pulmonary hypertension, neonatal disorders, risk factors, morbidity, mortality

## Abstract

**Background:** Pulmonary hypertension is one of the most common co-morbidities in infants with bronchopulmonary dysplasia (BPD), but its risk factors are unclear. The onset of pulmonary hypertension in BPD has been associated with poor morbidity- and mortality-related outcomes in infants. Two review and meta-analysis studies have evaluated the risk factors and outcomes associated with pulmonary hypertension in infants with BPD. However, the limitations in those studies and the publication of recent cohort studies warrant our up-to-date study. We designed a systematic review and meta-analysis to evaluate the risk factors and outcomes of pulmonary hypertension in infants with BPD.

**Objective:** To systematically evaluate the risk factors and outcomes associated with pulmonary hypertension in infants with BPD.

**Methods:** We systematically searched the academic literature according to the PRISMA guidelines across five databases (Web of Science, EMBASE, CENTRAL, Scopus, and MEDLINE). We conducted random-effects meta-analyses to evaluate the pulmonary hypertension risk factors in infants with BPD. We also evaluated the overall morbidity- and mortality-related outcomes in infants with BPD and pulmonary hypertension.

**Results:** We found 15 eligible studies (from the initial 963 of the search result) representing data from 2,156 infants with BPD (mean age, 25.8 ± 0.71 weeks). The overall methodological quality of the included studies was high. Our meta-analysis in infants with severe BPD revealed increased risks of pulmonary hypertension [Odds ratio (OR) 11.2], sepsis (OR, 2.05), pre-eclampsia (OR, 1.62), and oligohydramnios (OR, 1.38) of being small for gestational age (3.31). Moreover, a comparative analysis found *medium*-to-*large* effects of pulmonary hypertension on the total duration of hospital stay (Hedge's *g*, 0.50), the total duration of oxygen received (*g*, 0.93), the cognitive score (*g*, −1.5), and the overall mortality (g, 0.83) in infants with BPD.

**Conclusion:** We identified several possible risk factors (i.e., severe BPD, sepsis, small for gestational age, pre-eclampsia) which promoted the onset of pulmonary hypertension in infants with BPD. Moreover, our review sheds light on the morbidity- and mortality-related outcomes associated with pulmonary hypertension in these infants. Our present findings are in line with the existing literature. The findings from this research will be useful in development of efficient risk-based screening system that determine the outcomes associated with pulmonary hypertension in infants with BPD.

## Introduction

Bronchopulmonary dysplasia (BPD) is the most common chronic condition in pre-term newborns (1, 2). According to the American Lung Association, BPD is primarily a chronic pulmonary disorder that typically affects bronchial structures and causes alveolar sac dysplasia (3). Epidemiological studies have reported high worldwide BPD incidence rates of 10–89% in pre-term infants with low birth weight (2, 4, 5), and a global burden of disease study has reported a high mortality rate for infants with BPD (6).

Studies have suggested that proinflammatory cytokines increases (i.e., interleukin-1β, 6, 8, and tumor necrosis factor-α) together with vascular signaling changes (specifically in soluble fms-like tyrosine kinase-1, vascular endothelial growth factor, transforming growth factor β-1, and nuclear factor κ-light-chain-enhancer of activated B cells) between 24 and 36 weeks of gestation may be the primary underlying pathogenic mechanisms behind BPD (7–10). In addition, Jobe and Bancalari suggested that hyper-oxygenation may also play a role in the development of BPD through its pulmonary septation inhibition during the saccular phase (11). Together, these pathophysiological changes can promote fibrotic changes in alveolar septa and impair the pulmonary microvasculature, eventually leading to the development of BPD (7, 12, 13). The abnormal pulmonary microvasculature of infants with BPD leads to a range of cardiovascular manifestations (14) of which pulmonary hypertension is the most prominent (15, 16). De Paepe et al. (17) proposed a model in which re-modeling of the pulmonary microvasculature (i.e., reduced angiogenesis) together with modification in pulmonary vasoreactivity may promote resistance in the pulmonary vessels and precipitate the onset of pulmonary hypertension (12). The risks of pulmonary hypertension in infants with BPD are aggravated by several perinatal, growth-related, and demographic risk factors (18). Moreover, these risk factors may facilitate the onset of pulmonary hypertension in infants with BPD eventually worsening their morbidity and mortality-related outcomes (12). Despite the increased prevalence of pulmonary hypertension in infants with BPD, its impact and that of different risk factors on these infants is unclear. This type of evidence is necessary for clinicians to develop efficient risk stratification models to identify high-risk infants and improve their morbidity and mortality-related outcomes.

Many cohort studies have comparatively evaluated the risk factors and outcomes associated with pulmonary hypertension in infants with BPD (19–23). However, the role of certain risk factors on the development of pulmonary hypertension in infants with BPD remains unclear. For instance, some studies found a strong association of multiple births (24, 25) and pre-eclampsia (26, 27) with the development of pulmonary hypertension in infants with BPD. But, others found a weak association of these risk factors [multiple births (28, 29) and pre-eclampsia (30)] with the development of pulmonary hypertension. Similarly, the impact of pulmonary hypertension in infants with BPD on the morbidity and mortality-related outcomes is unclear, while some studies have reported a longer duration of hospital stay (27, 31) and longer oxygen supplementation requirements (19, 27, 28) in infants with BPD and pulmonary hypertension than in infants with BPD alone. While other studies found the opposite results (20, 21, 23, 32).

To the best of our knowledge, two systematic reviews and meta-analyses have separately evaluated the risk factors (18) and the outcomes (15), associated with pulmonary hypertension in infants with BPD. However, the findings from these studies have limitations because their authors failed to assess risk factors such as the presence of pre-eclampsia in the mothers and outcomes such as cognitive scores at the onset of pulmonary hypertension. Moreover, since the publication of those reviews, several high-quality cohort studies that evaluate the risk factors and outcomes associated with pulmonary hypertension in infants with BPD (21–23, 26, 29) have been published. Therefore, a synthesis of the updated knowledge through a systematic review and meta-analysis is strongly warranted.

In this systematic review and meta-analysis, we will attempt to clarify the evidence regarding risk factors and outcomes associated with pulmonary hypertension in infants with BPD. Our findings should be useful for pediatricians managing pulmonary hypertension in infants with BPD worldwide.

## Methods

We adhered to the PRISMA (Preferred Reporting Items for Systematic Reviews and Meta-Analyses) guidelines (33) for performing this meta-analysis.

### Data Search Strategy

We accessed the Web of Science, MEDLINE, CENTRAL, EMBASE, and Scopus databases to perform the literature search choosing the period from inception till January 2021. The search included a combination of MeSH keywords including “Bronchopulmonary dysplasia,” “BPD,” “pulmonary hypertension,” “risk factors,” “mortality,” “morbidity,” and “outcomes.” We also manually searched the bibliography section of the included studies to avoid missing relevant studies. We set out the following inclusion criteria:

a) Studies evaluating the risk factors associated with the development of pulmonary hypertension in infants with BPD.
b) Studies comparing the outcomes associated with pulmonary hypertension in infants with BPD
c) Studies with human participants
d) Case-control studies, prospective cohort trials, or retrospective cohort trials
e) Studies published in peer-reviewed scientific journals
f) Studies published in English.

Two reviewers independently performed the screening of the studies, and disagreements were resolved by discussion with a third independent reviewer.

### Quality Assessment

We appraised the risk of bias of the included studies using the Cochrane's risk of bias assessment tool for non-randomized controlled trials (34). This tool evaluates the outcomes for selective reporting, confounding bias, measurement of outcomes, and incomplete data availability as threats that can instigate bias. Two reviewers independently assessed the methodological quality of the studies included and, again, disagreements were solved after discussions with a third arbiter.

### Data Analysis

We performed the within-group meta-analysis using the Comprehensive Meta-analysis (CMA) version 2.0 software (35). We based our meta-analysis on the random-effects model (36). We calculated the odds ratios to identify risk factors (i.e., oligohydramnios, cesarean operation, sepsis, multiple births, small for gestational age, severe BPD, and pre-eclampsia) that led to the development of pulmonary hypertension in infants with BPD. We also evaluated weighted effect sizes, i.e., Hedge's *g* to compare the outcomes (i.e., cognition score, duration of hospital stay, duration of oxygen received, and mortality) in infants with BPD with or without pulmonary hypertension. The definition of BPD used in the included studies was in accordance with the 2001 NICHD criteria (37), moderate BPD was defined by the need for <30% supplemental oxygen and severe BPD as the need for ≥30% supplemental oxygen and/or positive pressure at 36 weeks post-menstrual age, which includes high flow nasal cannula. Besides, pulmonary hypertension in this study was defined as an abnormal growth of the pulmonary microvasculature and disruption of vascular in pre-term infants with BPD (23). Pulmonary hypertension in the included studies was diagnosed based on echocardiographic assessment, and defined as the presence of one or more of the following echo criteria: Right ventricular systolic pressure > 40 mmHg, bidirectional or right to left cardiac shunt, right ventricular systolic pressure /systemic systolic pressure >0.5, or interventricular septal systolic flattening with an eccentricity index more than 1.1 (38–40). We assessed the heterogeneity among studies by computing *I*^2^ statistics and classifying the heterogeneity as negligible (between 0 and 25%), moderate (between 25 and 75%), or substantial (≥75%) (41). We applied Duval and Tweedy's trim and fill procedure to evaluate the publication bias (42). This analysis is characterized by the imputation of studies from either side of the plotted graph to identify unbiased effects. We set the significance level for this study at 5%.

## Results

**Figure 1:**
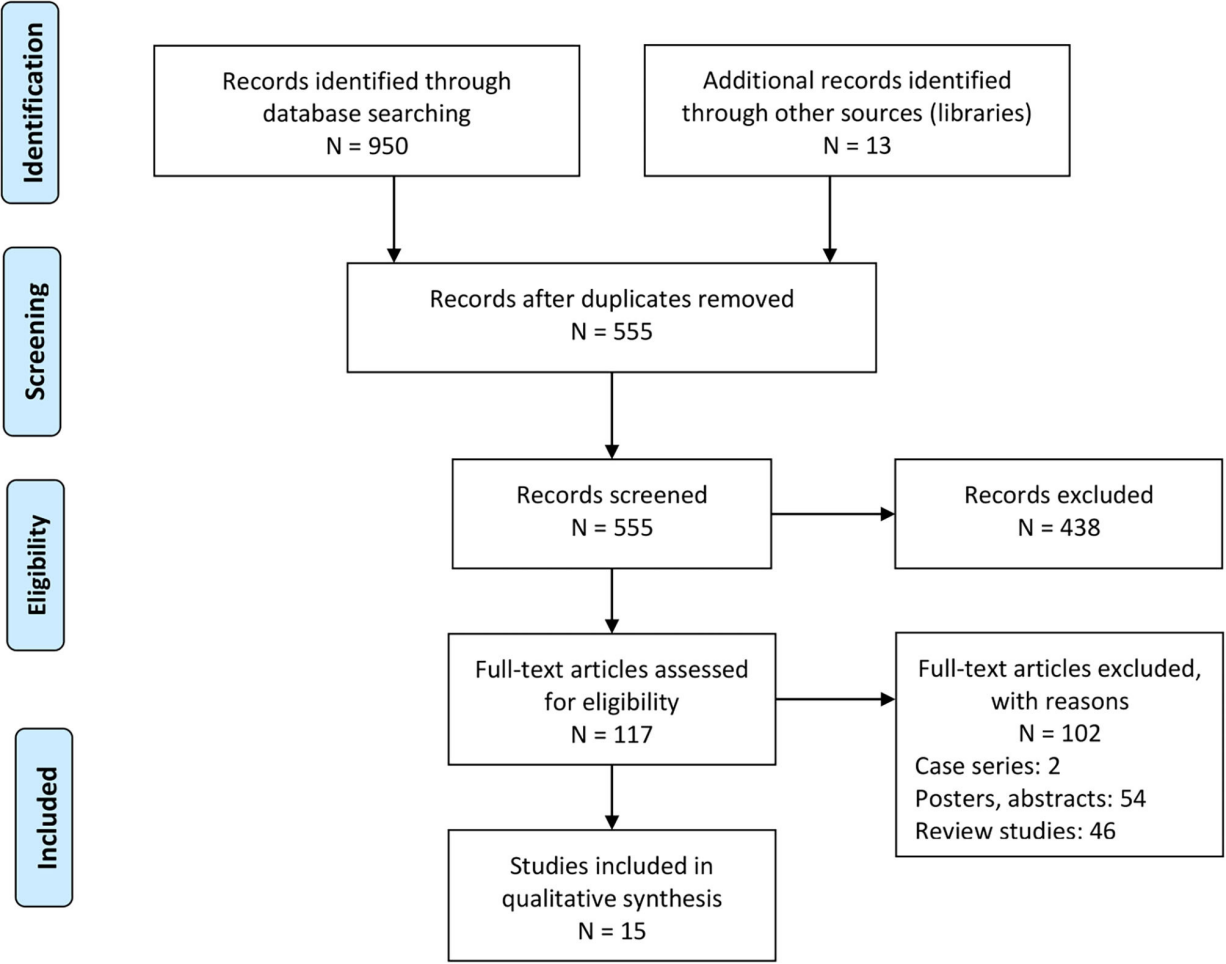
PRISMA flowchart.

Our academic literature search yielded a total of 950 studies. We identified an additional 13 during the manual screening of the reference sections of the included studies. After applying our inclusion criteria, we selected 15 studies. Of those, 12 were retrospective cohort studies (19–21, 24, 26–32, 43), 2 were prospective cohort studies (23, 25), and 1 was a case-control observational study (22) (Figure 1). We extracted the relevant data into tables, Table 1 shows a summary.

**Table 1:** Characteristics of the included studies.

**References**	**Country**	**Type of study**	**Sample descriptive**	**Age (M ± S.D weeks)**	**Birth weight (grams)**	**Risk factors (BPD vs. BPD-PH)**	**Outcomes (BPD vs. BPD-PH)**
MacKenzie et al. (21)	Canada	Retrospective cohort study	BPD: 63 (23F, 40M) BPD-PH: 24 (9F, 15M)	BPD: 26.4 ± 3.7BPD-PH: 24.7 ± 2.3	BPD: 840 ± 344 BPD-PH: 765 ± 220	Oligohydramnios: 9 vs. 1Cesarean: 42 vs. 10Sepsis: 20 vs. 9	Cognition score: 100 ± 15 vs. 90 ± 15 Hospital stay: 91 ± 40 vs. 99.5 ± 36 days Total duration oxygen received: 66 ± 45 vs. 81 ± 52.5 days Mortality: 2 vs. 0
Sheth et al. (22)	USA	Retrospective case-control observational study	BPD: 161 (59F, 102M) BPD-PH: 59 (32F, 27M)	BPD: 26 ± 12BPD-PH: 25 ± 4	BPD: 807 ± 206 BPD-PH: 690 ± 224	Oligohydramnios: 11 vs. 4Multiple birth: 41 vs. 15SGA: 12 vs. 16BPD severe: 115 vs. 56Cesarean: 112 vs. 39Pre-eclampsia: 34 vs. 15	Mortality: 7 vs. 10
Chen et al. (26)	China	Retrospective cohort study	BPD: 314 BPD-PH: 24	BPD: 27.2 ± 1.7BPD-PH: 27.1 ± 1.8	BPD: 1003 ± 240 BPD-PH: 798 ± 255	SGA: 24 vs. 8Sepsis: 23 vs. 13BPD severe: 23 vs. 16Pre-eclampsia: 6 vs. 3	-
Vayalthrikkovil et al. (23)	Canada	Prospective cohort study	BPD: 87 (43F, 44M) BPD-PH: 24 (11F, 13M)	BPD: 26.3 ± 1.5BPD-PH: 25.4 ± 1.8	BPD: 882 ± 223 BPD-PH: 729 ± 200	Cesarean: 57 vs. 15Oligohydramnios: 12 vs. 4SGA: 9 vs. 5Sepsis: 10 vs. 1BPD severe: 38 vs. 18	Hospital stay: 99 vs. 117 days Total duration oxygen received: 51 vs. 61 days
Choi et al. (29)	South Korea	Retrospective cohort study	BPD: 61 BPD-PH: 20	BPD: 25.8 ± 1.1BPD-PH: 25.3 ± 1.4	BPD: 758.6 ± 159.1 BPD-PH: 710.1 ± 183.6	Multiple birth: 35 vs. 3 Oligohydramnios: 3 vs. 4BPD severe: 23 vs. 17Sepsis: 15 vs. 11SGA: 9 vs. 5	Cognition score: 95 vs. 85 Hospital stay: 103 vs. 111 days
Mehler et al. (24)	Germany	Retrospective cohort study	BPD: 154 (74F, 80M) BPD-PH: 34 (18F, 16M)	BPD: 25BPD-PH: 25	BPD: 734 BPD-PH: 524	SGA: 10 vs. 6Multiple birth: 43 vs. 11BPD severe: 2 vs. 5	–
Kunjunju et al. (30)	Australia	Retrospective cohort study	BPD: 34 BPD-PH: 22	BPD: 25.8 ± 1.3BPD-PH: 25.8 ± 1.6	BPD: 770.2 ± 171 BPD-PH: 776.4 ± 226	Sepsis: 2 vs. 1Pre-eclampsia: 10 vs. 7	–
Nakanishi et al. (31)	Japan	Retrospective cohort study	BPD: 78 (41F, 37M) BPD-PH: 22 (11F, 11M)	BPD: 25.7 ± 1.4BPD-PH: 24.8 ± 1.3	BPD: 717 ± 181 BPD-PH: 702 ± 140	SGA: 37 vs. 11Sepsis: 23 vs. 8BPD severe: 13 vs. 15	Mortality: 0 vs. 1 Hospital stay: 147 vs. 170 days Cognitive score: 92 vs. 87
Choi et al. (20)	South Korea	Retrospective cohort study	BPD: 144 BPD-PH: 50	BPD: 26.6 ± 1.9BPD-PH: 26.2 ± 2	BPD: 825 ± 253 BPD-PH: 754 ± 227	Cesarean: 92 vs. 33SGA: 15 vs. 37	Total duration oxygen received: 86.3 vs. 104 days Hospital stay: 99.3 vs. 112 days Mortality: 0 vs. 3
Mourani et al. (25)	USA	Prospective cohort study	BPD: 238 (122F, 116M) BPD-PH: 39 (20F, 19M)	BPD: 26.3 ± 1.5BPD-PH: 25.4 ± 1.8	BPD: 922 BPD-PH: 810	Multiple birth: 54 vs. 16Cesarean: 176 vs. 34Sepsis: 39 vs. 7SGA: 38 vs. 9Pre-eclampsia: 62 vs. 12	Total duration oxygen received: 75 vs. 87 days Hospital stay: 90 vs. 90 Mortality: 3 vs. 4
Ali et al. (28)	Denmark	Retrospective cohort study	BPD: 57 (26F, 31M) BPD-PH: 17 (9F, 8M)	BPD: 26.9BPD-PH: 26.4	BPD: 924 BPD-PH: 836	Cesarean: 34 vs. 11Multiple birth: 19 vs. 2	Total duration oxygen received: 58 vs. 97 days
Check et al. (43)	USA	Retrospective cohort study	BPD: 99 (33F, 66M) BPD-PH: 39 (19F, 20M)	BPD: 25.5 ± 1.2BPD-PH: 26.3 ± 1.5	BPD: 862.9 ± 190.9 BPD-PH: 695.9 ± 190.9	Multiple birth: 31 vs. 5Sepsis: 33 vs. 18Oligohydramnios: 9 vs. 6Pre-eclampsia: 21 vs. 11	-
Kim et al. (27)	South Korea	Retrospective cohort study	BPD: 73 (29F, 44M) BPD-PH: 25 (8F, 17M)	BPD: 26.4 ± 1.9BPD-PH: 26.9 ± 2.4	BPD: 799 ± 234 BPD-PH: 765 ± 237	Sepsis: 23 vs. 9Pre-eclampsia: 9 vs. 7SGA: 7 vs. 7Cesarean: 49 vs. 19Multiple birth: 40 vs. 8	Hospital stay: 105.5 ± 29.8 vs. 147 ± 83.5 days Total duration oxygen received: 93.5 ± 28.4 vs. 141 ± 84.3 days
Slaughter et al. (32)	USA	Retrospective cohort study	BPD: 49 (21F, 28M) BPD-PH: 29 (15F, 14M)	BPD: 25 ± 1.4BPD-PH: 25 ± 1.5	BPD: 733 ± 144.1 BPD-PH: 735 ± 114.9	-	Total duration oxygen received: 113 ± 66.5 vs. 113 ± 56 days Mortality: 7 vs. 11
An et al. (19)	South Korea	Retrospective cohort study	BPD: 87 (33F, 54M) BPD-PH: 29 (1F, 28M)	BPD: 26.4BPD-PH: 26	BPD: 830 BPD-PH: 700	SGA: 26 vs. 14BPD severe: 18 vs. 25	Total duration oxygen received: 82 vs. 210 days

*M, Mean; SD, Standard deviation; F, Female; SGA, Small for gestational age; BPD, Bronchopulmonary dysplasia*.

### Participant Information

We gathered data from a total of 2,156 infants with BPD. Four studies failed to report the gender distribution in their samples (20, 26, 29, 30); therefore, we only know the total includes 657 female and 830 male infants The number of BPD cases with pulmonary hypertension was 457 (153 females, 188 males) and the number of BPD cases without pulmonary hypertension was 1,699 (504 females, 642 males).

The mean gestational age of all the infants was as 25.8 ± 0.71 weeks. In the group of infants with BPD and pulmonary hypertension, the mean age was 25.6 ± 0.76 weeks; and in the group of BPD without pulmonary hypertension, the mean age was 26.0 ± 0.63 weeks.

### Quality Assessment for Non-randomized Controlled Trials

**Figure 2:**
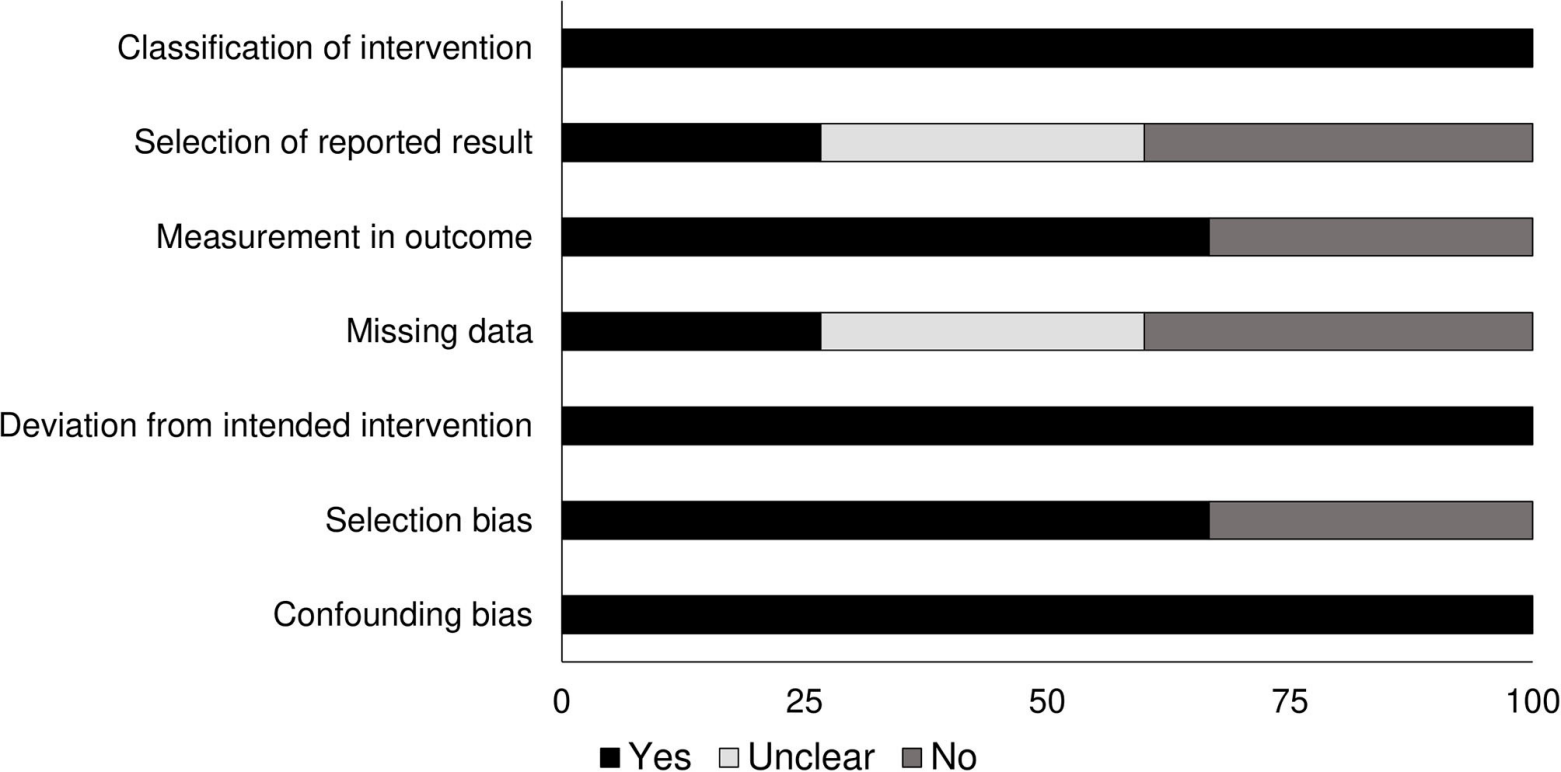
Risk of bias according to the Cochrane risk of bias assessment for the randomized controlled trials.

We analyzed the risk of bias in the methodology of the non-randomized controlled trials using the Cochrane's risk of bias assessment tool for non-randomized controlled trials. The overall risk of bias was low. Most cases of bias were due to missing data, selection of reported results, classification of intervention, measurement in the outcome, and selection bias. The graph on Figure 2 demonstrates the overall risk of bias.

### Publication Bias

**Figure 3:**
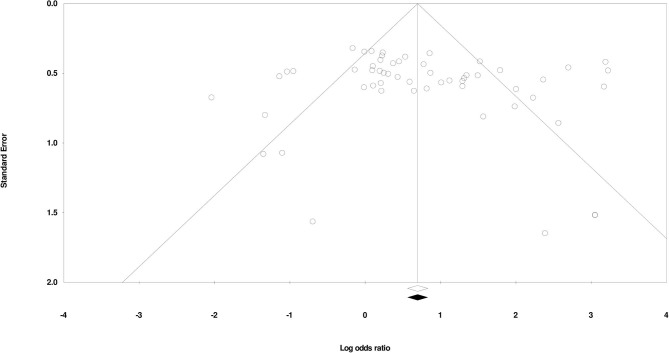
Publication bias by Duval and Tweedy's trim and fill method.

We used Duval and Tweedy's trim and fill method to determine missing studies according to the random effects model on either side of the mean effect of the funnel plot. We found no missing studies on either side of the mean effect. The overall random effects model determined the combined point estimate (2.13) and the 95% confidence interval (1.58–2.87) for all the studies (these values remained unchanged after application of the trim and fill method). Figure 3 shows the publication bias.

### Meta-Analysis Report

#### Risk Factors

##### Oligohydramnios

**Figure 4:**
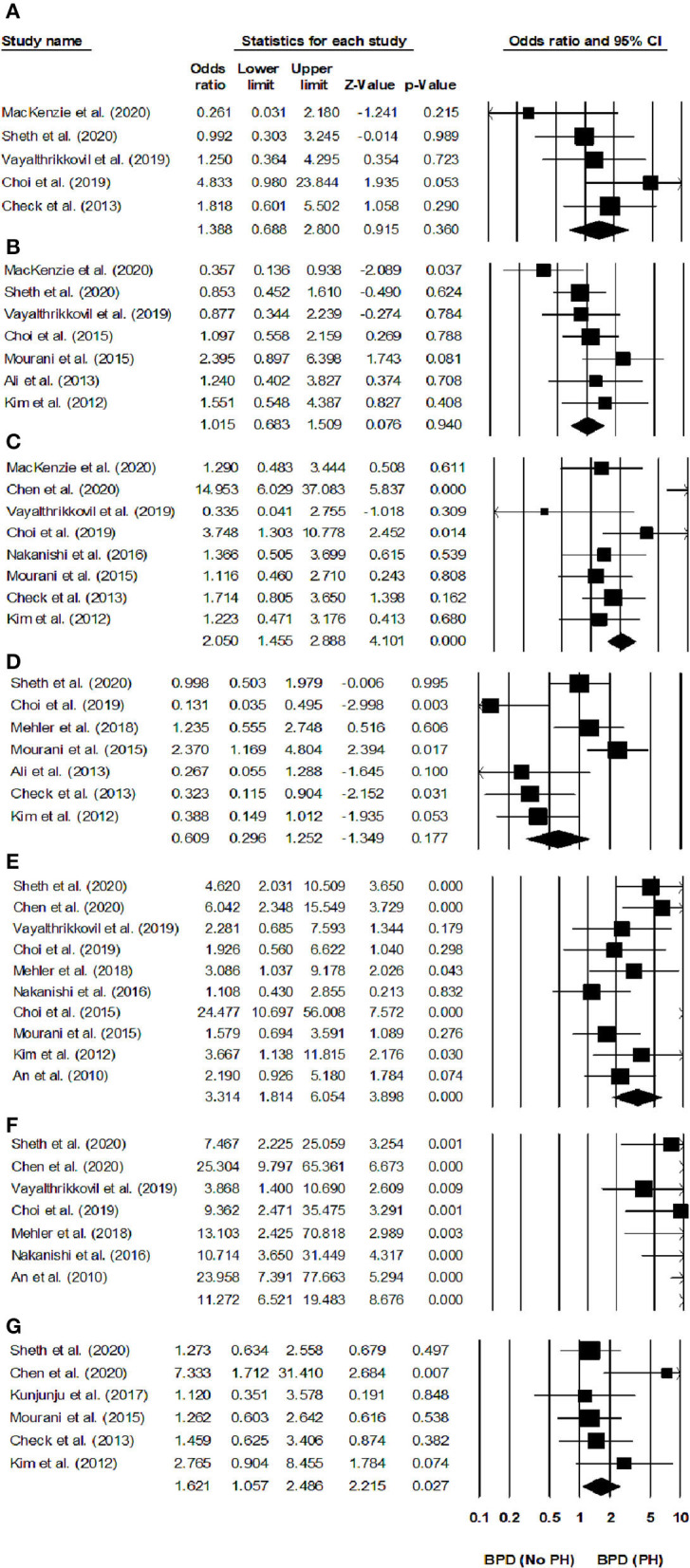
Forest plots of risk factors for developing pulmonary hypertension in infants with BPD. Forest plot for **(A)** oligohydramnios, **(B)** cesarean operation, **(C)** sepsis, **(D)** multiple births, **(E)** small for gestational age, **(F)** BPD severity, and **(G)** pre-eclampsia. The odds ratio is presented as black boxes, whereas 95% confidence intervals are presented as whiskers. A negative odds ratio represents a reduced risk for developing pulmonary hypertension in infants with BPD, a positive odds ratio represents an increased risk for developing pulmonary hypertension in infants with BPD.

We found five studies reporting the impact of oligohydramnios on the risk for developing pulmonary hypertension in infants with BPD (21–23, 29, 43). We observed an increased risk for developing pulmonary hypertension in infants with BPD and oligohydramnios (Figure 4A) (odds ratio, 1.38; 95% CI, 0.68–2.8; *p* = 0.36), with negligible study heterogeneity (*I*^2^, 8.9%).

##### Cesarean Section

Seven studies reported the impact of having been born by cesarean operation for developing pulmonary hypertension in infants with BPD (20–23, 25, 27, 28). We found that infants with BPD born by cesarean operation did not have a higher risk of developing pulmonary hypertension (Figure 4B) (odds ratio, 1.01; 95% CI, 0.68–1.50; *p* = 0.94), the study heterogeneity was negligible (*I*^2^, 5.6%).

##### Postnatal Sepsis

Eight studies evaluated the risk of pulmonary hypertension in infants with BPD and postnatal sepsis (21, 23, 25–27, 29, 31, 43). We found that postnatal sepsis increased the risk for developing pulmonary hypertension in infants with BPD (Figure 4C) (odds ratio, 2.05; 95% CI, 1.45–2.88, *p* < 0.001), with negligible study heterogeneity (*I*^2^, 5.6%).

##### Multiple Births

Seven studies evaluated the risk for pulmonary hypertension in multiple birth infants with BPD (22, 24, 25, 27–29, 43). We found that multiple birth infants with BPD had a similar risk of developing pulmonary hypertension than other infants with BPD (Figure 4D) (odds ratio, 0.60; 95% CI, 0.29–1.25; *p* = 0.17), with negligible study heterogeneity (*I*^2^, 5.9%).

##### Small for Gestational Age

Seven studies reported the risk of developing pulmonary hypertension in infants with BPD that were small for gestational age (22, 24, 25, 27–29, 43). We found that infants with BPD having a small weight for the gestational age had an increased risk of developing pulmonary hypertension than their normal-weight counterparts (Figure 4E) (odds ratio, 3.31; 95% CI, 1.81–6.05, *p* < 0.001), without study heterogeneity (*I*^2^, 0%).

##### Severe Bronchopulmonary Dysplasia

Seven studies assessed the risk of pulmonary hypertension in infants with severe BPD (19, 22–24, 26, 29, 31). We found that infants with severe BPD had an increased risk of pulmonary hypertension than other infants with BPD (Figure 4F) (odds ratio, 11.27; 95% CI, 6.52–19.48, *p* < 0.001), without study heterogeneity (*I*^2^, 0%).

##### Pre-eclampsia

Six studies assessed the risk of pulmonary hypertension in infants with BPD, whose mothers had had pre-eclampsia (22, 25–27, 30, 43). We found that pre-eclampsia increased the risk of developing pulmonary hypertension in infants with BPD (Figure 4G) (odds ratio, 1.62; 95% CI, 1.05–2.48; *p* = 0.02), with negligible study heterogeneity (*I*^2^, 8.6%).

### Outcomes

#### Duration of Hospital Stay

**Figure 5:**
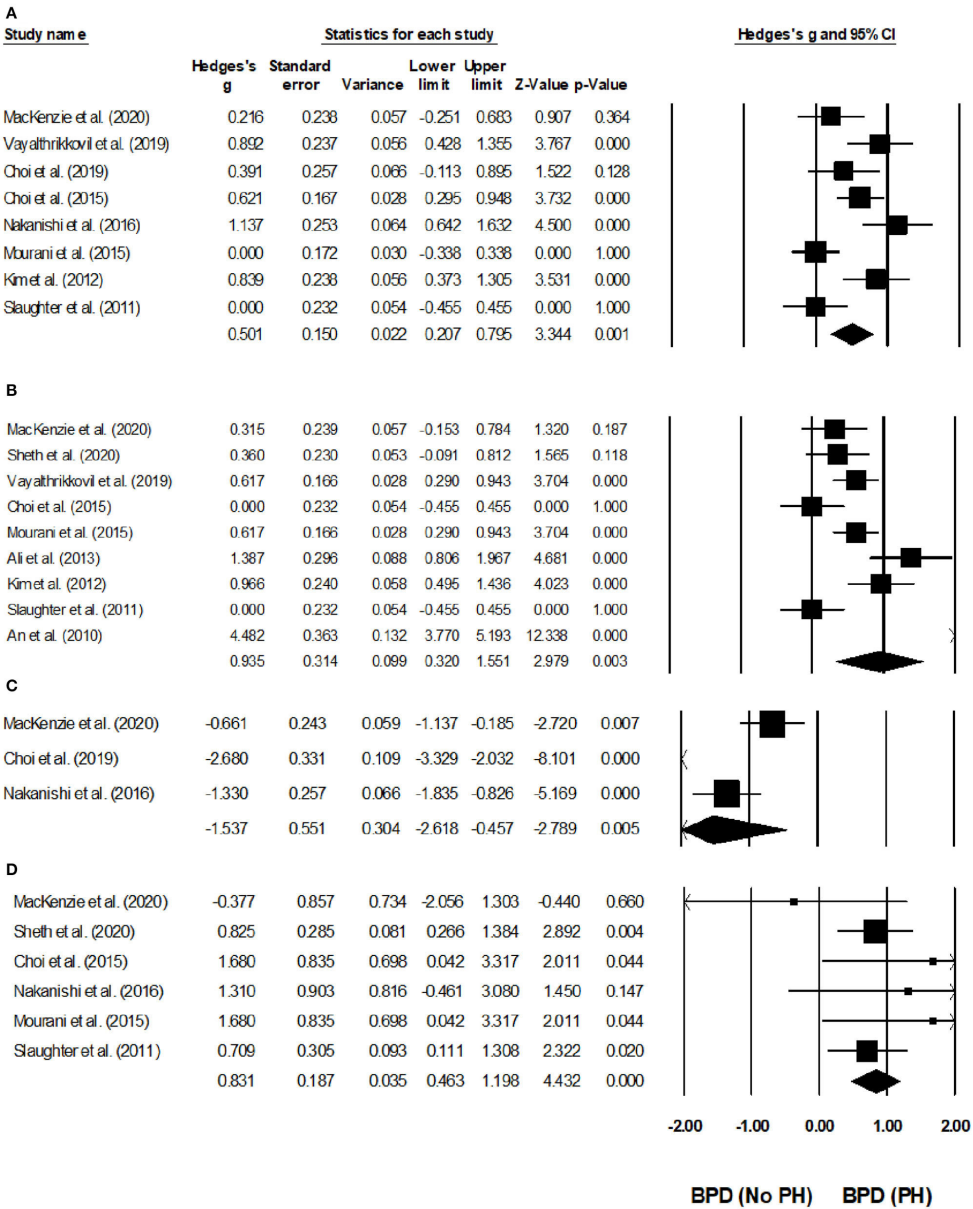
Forest plots for studies evaluating outcomes in infants with BPD infants with/without pulmonary hypertension. **(A)** Duration of hospital stay, **(B)** duration of oxygen received, **(C)** cognition score, **(D)** mortality. Weighted effect sizes are presented as black boxes, whereas 95% confidence intervals are presented as whiskers. A negative effect size represents a favorable outcome for infants with BPD without pulmonary hypertension, a positive effect size represents a favorable outcome for infants with BPD and pulmonary hypertension.

Eight studies reported the hospital stays of infants with BPD with and without pulmonary hypertension (20, 21, 23, 25, 27, 29, 31, 32). We found a *medium* positive effect on the hospital stays of infants with pulmonary hypertension as compared to that in infants without hypertension (Figure 5A) (Hedge's *g*, 0.50; 95% CI, 0.20–0.79; *p* = 0.001), with negligible heterogeneity (*I*^2^, 0.92%).

#### Duration of Supplementary Oxygen Needs

Nine studies assessed the duration of supplementary oxygen needs in infants with BPD with and without pulmonary hypertension (19–23, 25, 27–29, 31, 32). We observed a *large* positive effect in the supplementary oxygen need durations of infants with BPD infants with pulmonary hypertension as compared to BPD infants without hypertension (Figure 5B) (Hedge's *g*, 0.93; 95% CI, 0.32–1.55; *p* = 0.003), with moderate study heterogeneity (*I*^2^, 51.1%).

#### Cognition Score

Three studies reported the outcome of cognitive scores in infants with BPD infants with and without pulmonary hypertension (21, 29, 31). Two studies study reported that the cognitive testing was performed between 18 and 24 months of corrected age (21, 29), and one study reported that the cognitive testing was performed at 36 months of corrected age (20). We observed a *large* negative effect on the cognitive scores of infants with pulmonary hypertension as compared to that of infants without hypertension (Figure 5C) (Hedge's *g*, −1.5; 95% CI, −2.61 to −0.45, *p* < 0.01), with negligible study heterogeneity (*I*^2^, 12.7%).

#### Mortality

Six studies assessed the mortality of infants with BPD with or without pulmonary hypertension (20–22, 25, 31, 32). Two studies reported that they noted mortality outcomes before 36-week of post-menstrual age (20, 22), and one study each reported that they noted mortality outcomes after 36-week of post-menstrual age (31), and 12 months after birth (21). Two additional studies did not report the period when they observed the mortality outcomes (32, 44).

We observed a *large* positive effect on the mortality of infants with pulmonary hypertension as compared to that of infants without pulmonary hypertension (Figure 5D) (Hedge's *g*, 0.83; 95% CI, 0.46–1.19, *p* < 0.001), without study heterogeneity (*I*^2^, 0%).

## Discussion

The present systematic review and meta-analysis provides comprehensive evidence regarding the risk factors and outcomes associated with pulmonary hypertension in infants with BPD. We observed increased risks of pulmonary hypertension in infants with BPD and a severe disease presentation, in those small weight for gestational age, and in those with sepsis, pre-eclampsia history, and oligohydramnios. We also observed that pulmonary hypertension in infants with BPD led to poorer outcomes (manifested as longer hospital stays, longer supplementary oxygen needs, poorer cognitive scores, and higher mortality) than those in infants without pulmonary hypertension.

BPD management is considered one of the most challenging situation for pediatricians worldwide due to the disease's atypical pathophysiology, poor prognosis, and heterogeneous manifestations (45, 46). In addition to the pulmonary manifestations, infants with BPD present cardiovascular sequelae of which pulmonary hypertension is the most common one (11, 15). BDP's damage to the pulmonary microvasculature is thought to be the major pathophysiological mechanism precipitating the onset of pulmonary hypertension in these infants (11, 12). Vascular changes include the duplication of elastic laminae, medial-hyperplasia, and venous arterialization in the lungs; these changes increase pulmonary vascular resistance and eventually aggravate pulmonary hypertension (12). In severe cases of pulmonary hypertension, the high pulmonary vascular resistance impairs cardiovascular functioning and may lead to a reduced cardiac output and poor ventricular function that, in turn, impair oxygen delivery and instigate pulmonary edema (44). Together, pulmonary hypertension and BPD have been widely associated with poor short- and long-term morbidity and mortality outcomes (15, 18). Identifying pulmonary hypertension risk factors may help prevent the onset of this complication in infants with BPD (18).

In this systematic review, we identified risk factors that pre-dispose infants with BPD to pulmonary hypertension development. Primarily, perinatal, growth, and neonatal-intensive care unit-related factors (such as the presence of oligohydramnios, being small for gestational age, or having sepsis, a severe BPD presentation, a pre-eclampsia history, a mother with multiple births, or being born by cesarean section) are major risk factors for pulmonary hypertension in infants with BPD. For instance, in a cohort of 138 infants Check et al. (43) found that while pre-eclampsia (odds ratio, 0.6, 0.2–1.8) did not affect the risk of pulmonary hypertension in infants with BPD, oligohydramnios (OR, 1.6; CI, 0.5–5.3) did. Moreover, Choi et al. (20) identified sepsis (with a positive blood culture) as a risk factor for pulmonary hypertension in pre-term infants with severe BPD. They hypothesized that the presence of infection, especially in the hyper-oxygenated pulmonary tissues of infants with severe BPD, could promote an inflammatory response that would re-model the pulmonary vasculature leading to pulmonary hypertension (47). Some studies in our review identified severe BPD as an important risk factor for pulmonary hypertension. Mehler et al. (24) observed that most of the infants diagnosed with pulmonary hypertension had severe BPD (i.e., OR, 13; CI, 2–71). These authors also mentioned that the pulmonary hypertension in infants with severe BPD was more likely to be diagnosed in the hospital than after discharge, and that most treatments required immediate administration of sildenafil and supplemental oxygen. In this meta-analysis, we confirm these findings and report that a severe BPD (OR, 11.2; CI, 6.5–19.4), being small for gestational age (OR, 3.31; CI, 1.8–6.05), sepsis (OR, 2.05; CI, 1.4–2.8), pre-eclampsia history (OR, 1.62; CI, 1.05–2.4), and oligohydramnios (OR, 1.38; CI, 0.6–2.8) are all factors increasing the risk of pulmonary hypertension in infants with BPD. However, we did not find associations between being born by cesarean section (OR, 1.01; CI, 0.6–1.5) or from a mother with multiple births (OR, 0.60; CI, 0.29–1.2) and the risk of pulmonary hypertension in infants with BPD.

We synthesized the available evidence on the morbidity- and mortality-related outcomes of pulmonary hypertension in infants with BPD. In a prospective cohort with 111 infants with BPD, Vayalthrikkovil et al. (23) found poorer outcomes (longer hospital stays) in infants with pulmonary hypertension than in those without it. They associated the hospital stays with the dependence of the infants with pulmonary hypertension on respiratory support (i.e., invasive/non-invasive). Similarly, Nakanishi et al. (31) also found longer hospital stays and oxygen supplementation needs in infants with BPD and pulmonary hypertension than in those without it; and, they associated these effects with the presence of perinatal factors including intrauterine infections. In addition, they found histological chorioamnionitis and a high concentration of immunoglobulin M and in infants with pulmonary hypertension, but not in infants with BPD only. In our systematic review, we also found studies that had reported long-term implications of pulmonary hypertension in infants with BPD. Choi et al. (29), for instance, highlighted the neurodevelopmental sequelae of pulmonary hypertension and BPD among 81 infants mentioning that the infants with pulmonary hypertension had significantly poorer cognitive scores and growth deficits (i.e., body weight and head circumference) (*p* = 0.004) than the infants without pulmonary hypertension after 18–24 months of discharge. Likewise, we also observed that infants with BPD and pulmonary hypertension exhibited a higher mortality than their counterparts without pulmonary hypertension. in their cohort of 39 infants with pulmonary hypertension, Mourani et al. (25) reported that mortality events were often associated with the need for positive-pressure mechanical ventilation support. Barometric and volumetric trauma to the pre-mature pulmonary tissue due to ventilatory support may be the underlying reason for these increased mortality outcomes in infants with pulmonary hypertension (11, 48). In our meta-analysis, we confirmed these poor morbidity-related outcomes of longer hospital stays (Hedge's *g*, 0.50; CI, 0.2–0.79), longer supplementary oxygen needs (*g*, 0.93; CI, 0.3–1.5), and poorer cognitive scores (*g*, −1.5; CI, −2.6 to −0.4) in infants with pulmonary hypertension than in those without it. We also found poorer mortality outcomes (*g*, 0.83; CI, 0.4–1.19) for infants with pulmonary hypertension than for those without it.

Our systematic review and meta-analysis have a few limitations. First and foremost, we did not pre-register it in a systematic review repository such as the PROSPERO York or Joanna Briggs Institute one. We understand that this could raise concerns concerning the validity of our review (49). However, we assure our reviewers and our readers that we made several attempts to register the review, but failed to do so due to the current extended registration times at the repositories (longer than a year) owing to the COVID-19 pandemic crisis. Second, the paucity of data about cognition outcomes in the included studies may have influenced those results. We compared the cognition scores in BPD cases between those with/without pulmonary hypertension in three studies (21, 29, 31), but we cannot rule out a type II error in this regard due to the resulting small sample size (*n* = 268) we had to work with (50). Future studies should address these limitations with large cohorts comparing cognition outcomes in BPD cases with/without pulmonary hypertension.

In conclusion, our findings highlight the influence of variables such as having severe BPD, sepsis, pre-eclampsia, and oligohydramnios, or being small for gestational age toward the development of pulmonary hypertension in infants with BPD. Our results also show the negative impact of pulmonary hypertension on the total hospital stay length, the supplemental oxygen need length, the cognitive scores, and the overall mortality in infants with BPD. Our findings have implications for developing best practice guidelines for pre-emptively predicting and efficiently managing pulmonary hypertension in infants with BPD.

## Data Availability Statement

The raw data supporting the conclusions of this article will be made available by the authors, without undue reservation.

## Author Contributions

YC, DZ, and YL conceived and designed the study. AY and XW did literature search. XH, HS, and YD analyzed the data. YC, DZ, YL, and WZ wrote the paper. WZ reviewed and edited the manuscript. All authors read and approved the final manuscript.

## Conflict of Interest

The authors declare that the research was conducted in the absence of any commercial or financial relationships that could be construed as a potential conflict of interest.
